# Long noncoding RNA CYTOR sponges miR-195 to modulate proliferation, migration, invasion and radiosensitivity in nonsmall cell lung cancer cells

**DOI:** 10.1042/BSR20181599

**Published:** 2018-12-18

**Authors:** Jun Zhang, Wenqi Li

**Affiliations:** 1 Cancer Department, The People’s Hospital of China Three Gorges University, Yichang city, Hubei Province 443000, China; 2 Emergency Department, The First College of Clinical Medical Science, China Three Gorges University, Yichang city, Hubei Province 443002, China

**Keywords:** CYTOR, invasion, miR-195, radiotherapy

## Abstract

Nonsmall cell lung cancer (NSCLC) is one of the most frequent malignancies worldwide. Long noncoding RNAs (LncRNAs) play critical roles in cancer initiation and progression. Previous studies have demonstrated that overexpression of cytoskeleton regulator RNA (CYTOR) predicates poor prognosis and promotes tumor progression. However, the functional roles and underlying mechanism of CYTOR in NSCLC remain unknown. In the present study, we found that CYTOR promoted cell proliferation, migration and invasion ability, and induced radioresistance in NSCLC cells. Mechanistically, CYTOR could directly interact with miR-195 and increase its targets. Thus, CYTOR played an oncogenic role in NSCLC progression through sponging miR-195. Together, our study elucidates the role of CYTOR as a microRNA sponge in NSCLC, and CYTOR may be used as a promising therapeutic target for NSCLC treatment.

## Introduction

Lung cancer is the second most frequent malignancy worldwide and becoming the leading cause of cancer-related deaths in human [1]. Nonsmall cell lung cancer (NSCLC) accounts for approximately 85% of diagnosed lung cancer cases [2]. Despite intensive clinical efforts using multiple therapeutic approaches, the outcome of NSCLC patients is still unsatisfied. The overall 5-year survival rate remains at approximately 18% [3]. Thus, it is essential to reveal new detailed mechanisms and molecular pathways in NSCLC initiation and progression.

A large amount of human genome cannot encode proteins and the majority of transcripts are noncoding RNAs. Long noncoding RNAs (lncRNAs) are highly conserved transcripts consisting of larger than 200 nucleotides in length without protein-coding potential [4]. Emerging evidence has shown that lncRNAs play important roles in many cellular biological processes, such as cell proliferation, apoptosis, motility, autophagy and stemness [5–7]. Recently, it was found that lncRNAs are key regulators in various diseases including NSCLC [8–10]. For instance, lncRNA RHPN1-AS1 was observed to be down-regulated in gefitinib-resistant patients and associated with poor prognosis of NSCLC patients. RHPN1-AS1 sensitized gefitinib resistant in NSCLC cells. RHPN1-AS1 positively regulated the expression of TNFSF12 by association with miR-299-3p [11]. LncRNA SNHG20 was found to be up-regulated in NSCLC tissues and significantly associated with advanced tumor, lymph node and metastases (TNM) stage and tumor size, as well as poorer overall survival. SNHG20 interacted with EZH2 and then repressed P21 expression to accelerate proliferation and migration in NSCLC cells [12]. Recently, a novel lncRNA, cytoskeleton regulator RNA (CYTOR, also known as LINC00152), was functionally identified [13]. The aberrant expression of CYTOR was observed in some types of cancers, such as colorectal cancer [14], glioblastoma [15] and gallbladder cancer [16], in which it functioned as an oncogenic lncRNA. However, the biological role and detailed mechanism of CYTOR in NSCLC progression remain largely unknown.

In the present study, we demonstrated that CYTOR overexpression is a characteristic molecular change in NSCLC. CYTOR exerts its oncogenic activity through interaction with miR-195 in NSCLC. CYTOR is highly associated with NSCLC malignancies and may serve as a prognostic predictor for NSCLC patients in the clinic.

## Materials and methods

### Tissue samples

Sixty-four paired of tumoral and matched nontumoral tissue samples were obtained from the NSCLC patients underwent surgery at The People’s Hospital of China Three Gorges University from 2012 to 2017. Tissue samples were stored in liquid nitrogen until analysis. The present study was approved by the Ethics Review Committee of The People’s Hospital of China Three Gorges University and all the patients signed informed consents prior to the present study.

## Cell culture

Normal bronchial epithelial cells 16HBE and human NSCLC cell lines including A549, 95D, H358, H1299 and H1581 were purchased from Cellbank of Chinese Academy of Sciences (Shanghai, China). All the cells were cultured in DMEM (Gibco) with 10% fetal bovine serum (FBS, Gibco) in a humidified air with 5% CO_2_ at 37°C.

## Construction of stable cells with CYTOR knockdown

The shRNAs targeting CYTOR was inserted into pLKO.1 plasmid. The target sequences of CYTOR shRNAs were shown as follow: sh1: CTGGAAACCTCTTGACTCT; sh2: CAGGAAGCTCTATGACACA. Scramble shRNA was taken as control (shNC). The lentivirus particles were packaged in 293T cell. The lentivirus was harvested and then infected A549 and 95D cells. The stable A549 and 95D cells were screened by using puromycin for 2 weeks.

## Construction of stable cells with CYTOR overexpression

Full-length CYTOR cDNA was amplified from cDNA of A549 cells and cloned into pcDNA3.1 plasmid (pcDNA-CYTOR). The pcDNA3.1 empty vector was used as the control (pcDNA-NC). H1299 and H1581 cells were transfected with pcDNA-NC or pcDNA-CYTOR using Lipofectamine 3000 (Invitrogen). After 48 h, stable H1299 and H1581 cells were selected by using G418 for 2 weeks.

## RNA extraction and quantitative real-time PCR

Total RNA from the tissues and cells was extracted by using TRIZOL regent (Invitrogen) according to the standard protocol and then reversely transcribed to complementary DNA (cDNA) using SuperScript First Strand cDNA System (Invitrogen). Then quantitative real-time PCR (qRT-PCR) analyses were performed with a SYBR Premix Ex Taq kit (Takara, Dalian, China) on an ABI StepOne real-time PCR system (Applied Biosystems). The relative expression levels of target genes were calculated using 2^−ΔΔ*C*^_T_ method and normalized to GAPDH. The primers used were shown as follows: CYTOR: 5′-AGAATGAAGGCTGAGGTGTG-3′ (forward) and 5′-CAGCGACCATCCAGTCATTTA-3′ (reverse); YAP: 5′-CAGACAGTGGACTAAGCATGAG-3′ (forward) and 5′-CAGGGTGCTTTGGTTGATAGTA-3′ (reverse); GDPD5: 5′-CAGCGCTCCCATGATGATAC-3′ (forward) and 5′-GGACTTCCCACCAGAAGTAAAG-3′ (reverse); WNT3A: 5′-GACTTCCTCAAGGACAAGTACG-3′ (forward) and 5′-GGCACCTTGAAGTAGGTGTAG-3′ (reverse); CARM1: 5′-CCGCGGTGGATGAGTATTT-3′ (forward) and 5′-ACCGTGTACTTGACAGACTTG-3′ (reverse).

## CCK-8 assay

Cell counting kit-8 (CCK-8) assay was adopted to evaluate the cell proliferation. Briefly, cells were seeded into 96-well plates at the density of 2000 cells/well. At different time point, cells were incubated with 10 μl of CCK-8 for 1.5 hs at 37°C. The optical density (OD) at 450 nm was measured using a Microplate Reader (Biorad).

### microRNA transfection

For microRNA transfection, miR-195 mimics or miR-negative control (miR-NC) mimics or miR-195 inhibitor (inh-195) or miR-NC inhibitor (inh-NC) was transfected into indicated NSCLC cells using lipofectamine 3000 (Invitrogen) for 48 h and then used for following detection.

## Migration and invasion assay

Migration assay was performed in 24-well inserts (8-μm pore size; Corning), according to manufacturer’s instructions. About 1.0 × 10^5^ cells were seeded in serum-free medium in the top chamber of a Transwell, while the DMEM media containing 20% FBS were placed in the lower chamber. For the invasion assay, Matrigel (Corning, U.S.A.) was diluted with serum-free DMEM medium and then plated into the upper chamber of an insert with an 8-μm pore size (Corning, U.S.A.). About 2 × 10^5^ cells were seeded into the upper chamber for invasion assays, and DMEM medium with 20% FBS was added into the lower chamber. After incubation for 24 h, cells remaining in the upper chamber were wiped off, and cells on the lower chamber were fixed with 4% paraformaldehyde and stained with Crystal Violet. The numbers of cells that migrated or invaded were counted in five different fields with a microscope.

## Ionizing radiation (IR) treatment of cells

Cells were irradiated by a linear accelerator (Varian Medical Systems, U.S.A.) in 25-T flasks. Cells were exposed to varying doses of radiation treatment (0, 2, 4, 6 and 8 Gy) with a 6-MV photon beam at a dose rate of 3.5 Gy/min and 160 kv X-ray energy. After 48 h, cells were used for further analysis.

## Apoptosis assay

Forty-eight hours after IR treatment, the cells were harvested and washed with phosphate-buffered saline (PBS). The cell apoptosis detection was performed by using Apoptosis Detection Kit (Dojindo) according to the manufacturer’s instruction. The samples were analyzed using a FACScan flow cytometer (Becton Dickinson; San Jose, CA, U.S.A.). Each assay was performed in triplicate.

## Luciferase reporter assay

CYTOR containing the predicted wild-type or mutant (mut) miR-195 binding sites were cloned into pmirGLO plasmid (Promega, Madison, WI, U.S.A.). The recombinant plasmids were designated as pmirGLO-CYTOR and pmirGLO-CYTOR-mut. For luciferase activity assay, NSCLC cells were co-transfected with pmirGLO-CYTOR or pmirGLO-CYTOR-mut and miR-195 or miR-NC by Lipofectamine 3000 (Invitrogen) according to the manufacturer’s instructions. After 24 h, the luciferase activity was measured using the Dual-Luciferase Reporter Assay system (Promega). Firefly luciferase activity was normalized to the corresponding Renilla luciferase.

## Isolation of cytoplasmic and nuclear RNA

Cytoplasmic and nuclear RNAs were isolated and purified using the Cytoplasmic & Nuclear RNA Purification Kit (Norgen, Belmont, CA) according to the manufacturer’s instructions.

## RNA immunoprecipitation (RIP) assay

RIP assay was performed as previously described [17]. NSCLC cells were co-transfected with pcDNA-MS2, pcDNA-CYTOR-MS2, pcDNA-CYTOR-mut-MS2 and pMS2-GFP (pMS2-GFP was a gift from Robert Singer, Departments of Anatomy and Structural Biology and Cell Biology, Albert Einstein College of Medicine Bronx (Addgene plasmid # 27121 [18]). After 48 h, cells were used to perform RIP assay using a GFP antibody (Abcam) and the Magna RIP™ RNA-Binding Protein Immunoprecipitation Kit (Millipore, Bedford, MA) according to the manufacturer’s instructions. For anti-AGO2 RIP, NSCLC cells were transfected with miR-195 or miR-NC mimics. After 48 h, cells were used to perform RIP experiments using an AGO2 antibody (Millipore) as described above.

## RNA pull-down

CYTOR and CYTOR-mut were *in vitro* transcribed respectively from vector pSPT19-CYTOR and pSPT19-CYTOR-mut, and biotin-labeled with the Biotin RNA Labeling Mix (Roche) and T7 RNA polymerase (Roche), treated with RNase-free DNase I (Roche), and purified with an RNeasy Mini Kit (Qiagen, Valencia, CA). About 1 mg of whole-cell lysates from NSCLC cells were incubated with 3 μg of purified biotinylated transcripts for 1 h at 25°C; complexes were isolated with streptavidin agarose beads (Invitrogen). The RNA present in the pull-down material was detected by qRT-PCR analysis.

## Statistical analysis

Data were expressed as mean ± SD. Student’s *t-*test or one-way analysis of variance was used to determine statistical differences. The association between CYTOR and miR-195 was analyzed by Pearson correlation test. A *P*-value less than 0.05 was considered statistically significant.

## Results

### CYTOR is up-regulated in NSCLC tissues and indicates poor prognosis

**Figure 1:**
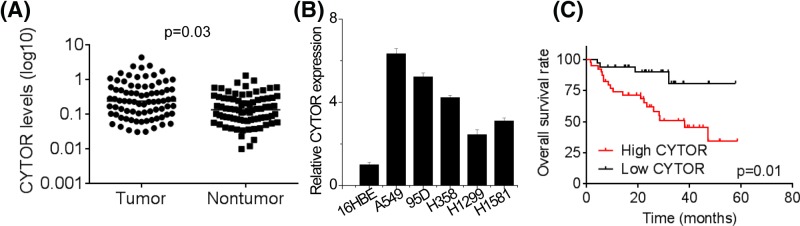
CYTOR is up-regulated in NSCLC tissues and cells and indicates poor prognosis (**A**) CYTOR expression was analyzed by qRT-PCR in NSCLC and adjacent nontumoral tissues. (**B**) Expression of CYTOR in NSCLC cell lines and normal bronchial epithelial cells (16HBE) was analyzed by qRT-PCR. (**C**) Using the median of CYTOR expression in NSCLC tissues as the threshold, NSCLC patients were divided into low expression group and high expression group. The expression of CYTOR in NSCLC tissues negatively correlates with patient overall survival rate. Using the Kaplan–Meier analysis, patients with higher expression level of CYTOR showed a worse overall survival than the patients with lower expression level of CYTOR.

In order to explore the clinical significance of CYTOR expression in NSCLC patients, qRT-PCR analysis was used to measure the expression levels of CYTOR in 64 pairs of tumoral and matched nontumoral tissues. As shown in Figure 1A, CYTOR expression was significantly increased in NSCLC tissues relative to adjacent nontumorous tissues. According to clinicopathological feature analysis (Table 1), higher CYTOR expression was observed more frequently in patients with larger tumor size (*P *= 0.011), advanced TNM stage (*P*= 0.024) and lymph node metastasis (*P* = 0.024). Nevertheless, other parameters including gender (*P*=0.611), age (*P*=0.451), smoking status (*P*=0.451) and differentiation grade (*P*=0.313) were not correlated with CYTOR expression. Similarly, it was found that CYTOR levels in NSCLC cell lines were increased compared with normal bronchial epithelial cells 16HBE (Figure 1B). Moreover, Kaplan–Meier overall survival (OS) analysis was performed to explore whether CYTOR could be regarded as a prognostic predictor for NSCLC patients. The results showed that the OS of the patients with higher CYTOR expression was significantly shorter than that of the patients with lower CYTOR expression (Figure 1C). These data suggest that CYTOR may play a critical role in the progression of NSCLC.

**Table 1:** Relationship between CYTOR expression and clinicopathological characteristics of NSCLC patients

Parameter	CYTOR expression		*P* value
	Low	High	
Gender			
Male	18	20	0.611
Female	14	12	
Age (years)			
≥60	19	16	0.451
<60	13	16	
Smoking			
Ever	16	19	0.451
Never	16	13	
Differentiation			
Well	16	12	0.313
Moderate/poor	16	20	
Tumor size			
≥4 cm	8	18	0.011
<4 cm	24	14	
TNM			
I-II	19	10	0.024
III	13	22	
Lymph node metastasis			
No	21	12	0.024
Yes	11	20	

Using the median of CYTOR expression in NSCLC tissues as the threshold, NSCLC patients were divided into low expression group and high expression group.

### CYTOR promotes the proliferation, migration and invasion of NSCLC cells

**Figure 2:**
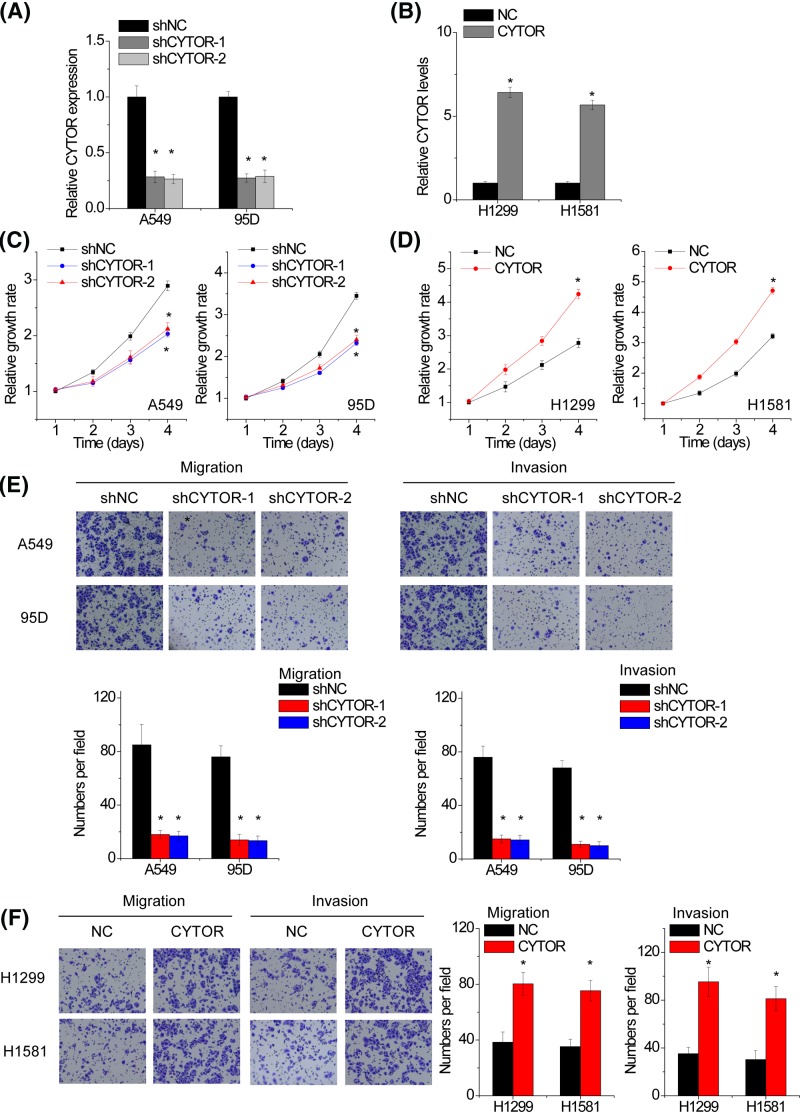
CYTOR promotes the proliferation, migration and invasion of NSCLC cells (**A**) qRT-PCR analysis of lncRNA CYTOR expression in negative control (shNC) and CYTOR-silencing (shCYTOR) A549 and 95D cells. (**B**) qRT-PCR analysis of lncRNA CYTOR expression in control (NC) and CYTOR-overexpressing (CYTOR) H1299 and H1581 cells. (**C**) CCK-8 assay was performed to detect cell proliferation of control or shCYTOR transfected A549 and 95D cells. (**D**) CCK-8 assay was performed to detect cell proliferation of control or CYTOR transfected H1299 and H1581 cells. (**E**) Transwell assay was performed to detect cell migration and invasion of control or shCYTOR transfected A549 and 95D cells. (**F**) Transwell assay was performed to detect cell migration and invasion of control or CYTOR transfected H1299 and H1581 cells. Significantly different from control (*P<0.05).

We next explored the functional significance of CYTOR in NSCLC cells. Because A549 and 95D have the highest levels of CYTOR among all the NSCLC cell lines, two shRNAs specifically targeting CYTOR were used to knockdown CYTOR expression in A549 and 95D cells (Figure 2A). We overexpressed CYTOR in H1299 and H1581 cells due to their lower levels of CYTOR (Figure 2B). CCK-8 assays were performed to detect the effect of CYTOR on cell proliferation. The results showed that the proliferative ability was significantly attenuated after CYTOR silence in both A549 and 95D cells (Figure 2C). Conversely, CYTOR overexpression accelerated the proliferation of H1299 and H1581 cells compared with control group (Figure 2D). Moreover, transwell assay demonstrated that CYTOR depletion dramatically inhibited the migration and invasion ability of A549 and 95D cells (Figure 2E). In contrast, ectopic overexpression of CYTOR enhanced migratory and invasive capacity in H1299 and H1581 cells (Figure 2F).

### CYTOR suppresses the radiosensitivity of NSCLC cells

**Figure 3:**
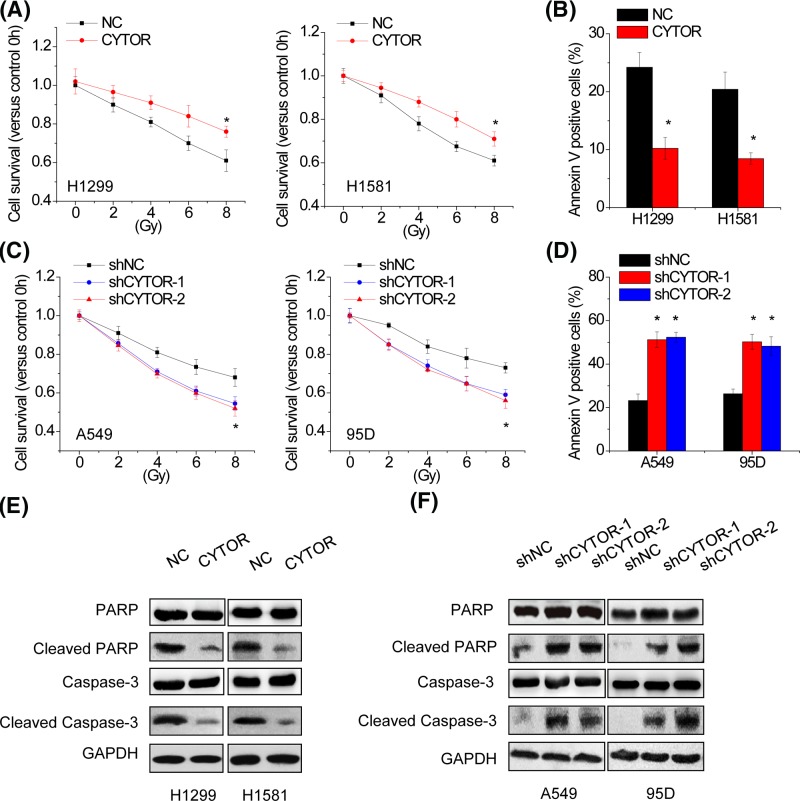
CYTOR suppresses the radiosensitivity of NSCLC cells (**A**) Control and CYTOR overexpressing H1299 and H1581 cells were exposed to varying doses of radiation (0, 2, 4, 6, and 8 Gy). CCK-8 assay was used to determine the cell viability 48 h after irradiation (IR). Cell viability is expressed as the percentage relative to the control at 0 Gy. (**B**) H1299 and H1581 cells with or without CYTOR overexpression were subjected to 8 Gy radiation. Cell apoptosis was assessed by staining with annexin V and propidium iodide 48 h after IR. The percentage of apoptotic cells was determined using flow cytometric analysis. (**C**) Control and CYTOR knockdown A549 and 95D cells were exposed to varying doses of radiation (0, 2, 4, 6 and 8 Gy). CCK-8 assay was used to determine the cell viability 48 h after IR. Cell viability is expressed as the percentage relative to the control at 0 Gy. (**D**) A549 and 95D cells with or without CYTOR knockdown were subjected to 8 Gy radiation. Cell apoptosis was assessed by staining with annexin V and propidium iodide 48 h after IR. The percentage of apoptotic cells was determined using flow cytometric analysis. (**E**) Western blot analysis showing the expression levels of total and cleaved Caspase-3 and PARP following CYTOR overexpressing in H1299 and H1581 cells. (**F**) Western blot analysis showing the expression levels of total and cleaved Caspase-3 and PARP following CYTOR silencing in A549 and 95D cells. Significantly different from control (*P<0.05).

We further determined whether CYTOR could affect the radiosensitivity of NSCLC cells. H1299 and H1581 with CYTOR overexpression cells were exposed to irradiation (IR) treatment. Cell viability assessment by CCK-8 assay showed that IR decreased the cell viability in a dose-dependent manner, whereas CYTOR overexpression suppressed the loss of cell viability by IR in both H1299 and H1581 cells (Figure 3A). Further apoptosis analysis showed that IR induced apoptosis in a dose-dependent manner, whereas CYTOR overexpression significantly inhibited IR-induced apoptosis in H1299 and H1581 cells (Figure 3B). Conversely, CYTOR knockdown enhanced the loss of cell viability and the apoptosis by IR in A549 and 95D cells (Figure 3C,D). Consistent with above observation, the expression levels of well-defined apoptosis protein markers, including cleaved PARP and cleaved caspase 3, markedly decreased in H1299 and H1581 cells with CYTOR overexpression, whereas increased in A549 and 95D cells with silenced CYTOR expression (Figure 3E,F). Together, these results suggest that CYTOR suppresses radiosensitivity of NSCLC cells.

### CYTOR is physically associated with miR-195

**Figure 4:**
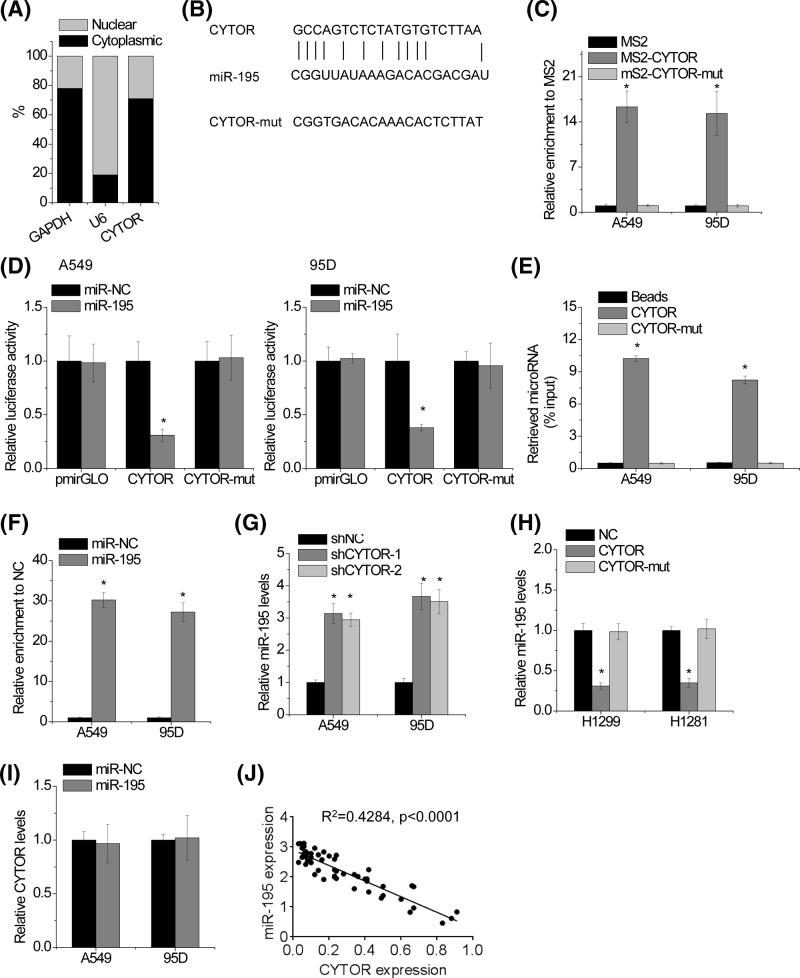
CYTOR is physically associated with miR-195 (**A**) Fractionation of A549 cells followed by qRT-PCR. GAPDH and U6 were used as endogenous controls. (**B**) Schematic outlining the predicted binding sites of miR-195 on CYTOR. (**C**) MS2-RIP followed by microRNA qRT-PCR to detect microRNAs endogenously associated with CYTOR. (**D**) Luciferase activity in A549 and 95D cells co-transfected with miR-195 and luciferase reporters containing nothing, CYTOR or mutant transcript (CYTOR-mut). Data are presented as the relative ratio of firefly luciferase activity to renilla luciferase activity. (**E**) A549 and 95D cell lysates were incubated with biotin-labeled CYTOR; after pull-down, microRNAs were extracted and assessed by qRT-PCR. (**F**) Anti-AGO2 RIP was performed in A549 and 95D cells transiently overexpressing miR-195, followed by qRT-PCR to detect CYTOR associated with AGO2. (**G**) The miR-195 expression in control and CYTOR knockdown A549 and 95D cells was detected by qRT-PCR. (**H**) The miR-195 expression in control and CYTOR overexpressing H1299 and H1281 cells was detected by qRT-PCR. (**I**) The CYTOR expression in A549 and 95D cells transfected with miR-195 mimics was detected by qRT-PCR. (**J**) The correlation between CYTOR transcript level and miR-195 level in 62 NSCLC tissues was analyzed by Pearson correlation analysis. Significantly different from control (*P<0.05).

We found that CYTOR mainly located in the cytoplasm (Figure 4A). As cytoplasmic lncRNAs are known to function as microRNAs sponge and induce the inhibition of target microRNAs activities [19], suggesting that CYTOR may exert its function in this manner. The TargetScan prediction algorithm identified tumor-suppressing miR-195 as target microRNAs of CYTOR (Figure 4B). We then explored whether CYTOR functions as a sponge of miR-195. To validate the direct interaction between CYTOR and miR-195 at the endogenous level, a MS2-RIP was used to pull-down endogenous microRNAs associated with CYTOR. As shown in Figure 4C, the CYTOR RIP was significantly enriched for miR-195 compared with those of the empty vector (MS2), IgG and CYTOR with mutated miR-195 targeting sites (CYTOR-mut) in A549 and 95D cells. In addition, luciferase reporters containing either wild-type or mutant CYTOR were constructed. Overexpression of miR-195 mimics decreased the luciferase activity of the wild-type reporter vector, but not of the empty vector or the mutant CYTOR reporter vector (Figure 4D). Specific interaction between miR-195 and CYTOR was further confirmed by affinity pull-down of endogenous miR-195 using *in vitro*-transcribed biotin-labeled CYTOR (Figure 4E). It is known that microRNAs bind to their targets and induce translational repression and/or mRNA degradation in an AGO2-dependent manner [20]. To determine whether CYTOR is regulated by miR-195 in this manner, anti-AGO2 RIP was performed in A549 and 95D cells transfected with miR-195. It was found that CYTOR was specifically pulled down by anti-AGO2 antibody in miR-195-overexpressing cells, suggesting that miR-195 is a bona fide CYTOR-target microRNA binding to CYTOR (Figure 4F). Knockdown of CYTOR increased miR-195 expression in A549 and 95D cells (Figure 4G), whereas overexpression of CYTOR decreased miR-195 expression in H1299 and H1581 cells (Figure 4H). However, we found no significant difference in CYTOR levels after transfection of miR-195 mimics (Figure 4I). These data revealed that miR-195 physically binds to CYTOR but does not induce the degradation of CYTOR. We next examined the pathological association between CYTOR and miR-195 in human NSCLC samples. It was observed that CYTOR transcript level was significantly negatively correlated with miR-195 level (Figure 4J). All these results suggest an important role of CYTOR in suppression of miR-195.

### CYTOR up-regulates the miR-195 targets

**Figure 5:**
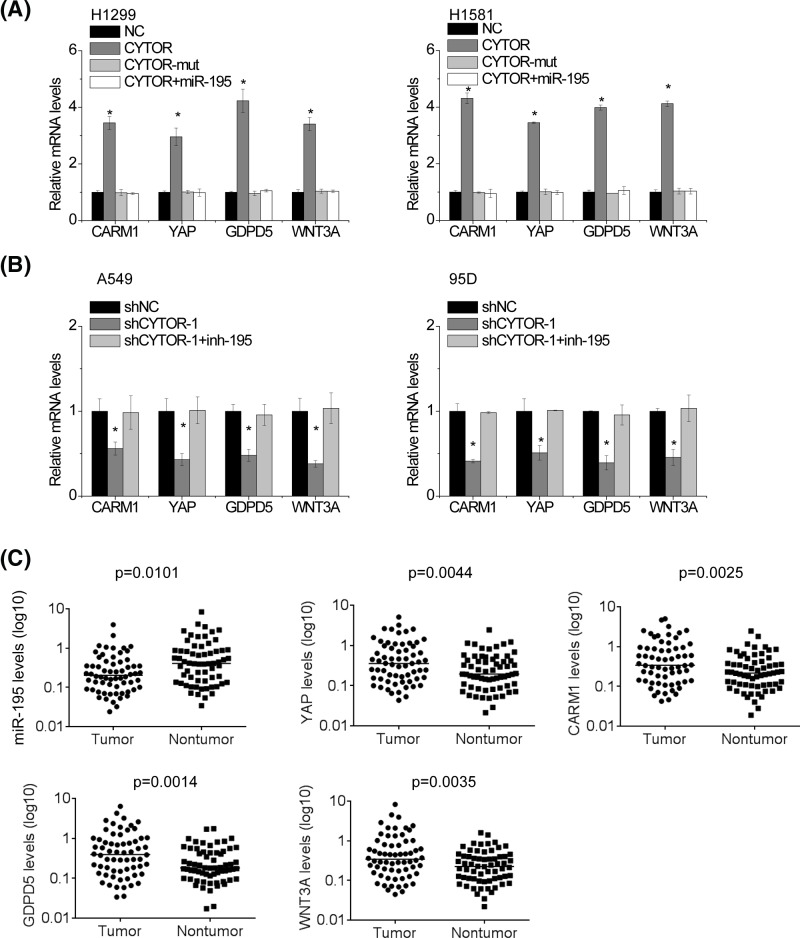
CYTOR up-regulates the miR-195 targets (**A**) The mRNA levels of CARM1, YAP, GDPD5 and WNT3A in indicated H1299 and H1581 cells. (**B**) The mRNA levels of CARM1, YAP, GDPD5 and WNT3A in indicated A549 and 95D cells. (**C**) The expression levels of indicated genes in 64 pairs of NSCLC and adjacent nontumoral tissues were examined by qRT-PCR. Significantly different from control (*P<0.05).

It has been reported that miR-195 suppresses the proliferation, migration, invasion and radiosensitivity of cancer cells through directly targeting some oncogenes, including CARM1, YAP, GDPD5 and WNT3A [21–24]. Because CYTOR shares regulatory miR-195 with CARM1, YAP, GDPD5 and WNT3A, we examined whether CYTOR could modulate these miR-195 targets. The miR-195 target genes CARM1, YAP, GDPD5 and WNT3A were up-regulated after overexpression of CYTOR in H1299 and H1581 cells, but not the mutant (Figure 5A). Ectopic expression of miR-195 abrogated this increase. Additionally, we inhibited miR-195 expression in CYTOR silencing NSCLC cells. Knockdown of CYTOR decreased CARM1, YAP, GDPD5 and WNT3A expression in both A549 and 95D cells. Silence of miR-195 overcame this decrease (Figure 5B). In addition, we detected miR-195, CARM1, YAP, GDPD5 and WNT3A in 64 pairs of NSCLC and adjacent nontumor tissues. Our data showed that miR-195 expression levels were significantly decreased in NSCLC tissues, while CARM1, YAP, GDPD5 and WNT3A mRNA levels were markedly increased in NSCLC tissues (Figure 5C).

### CYTOR exerts oncogenic activity through suppression of miR-195

**Figure 6:**
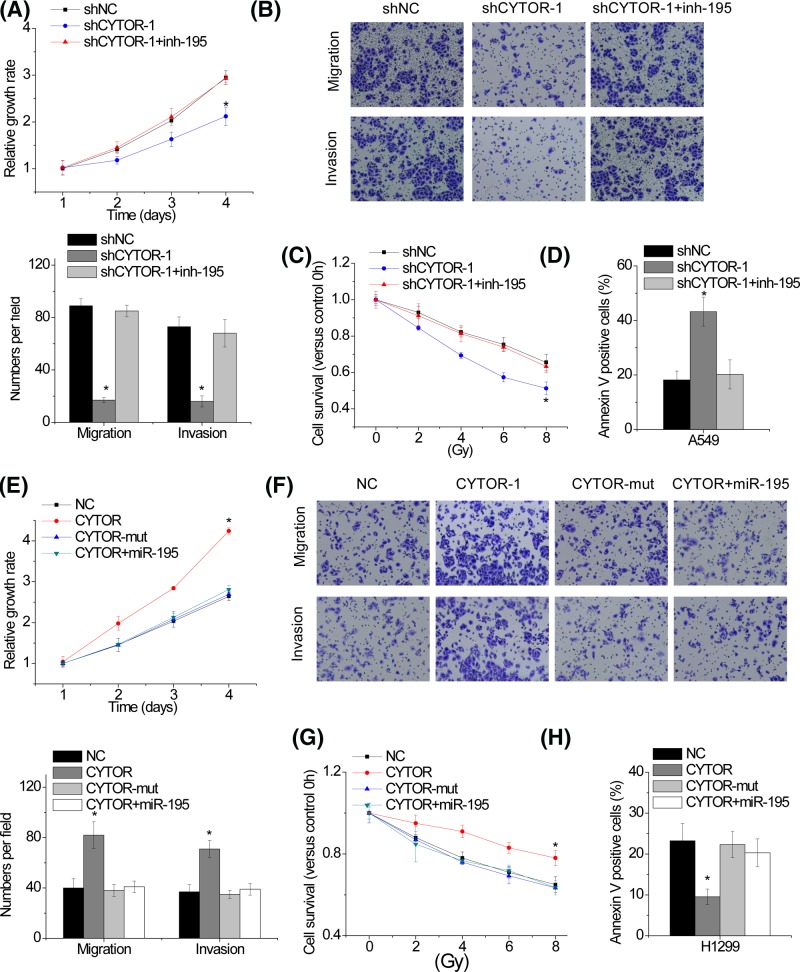
CYTOR exerts oncogenic activity through suppression of miR-195 (**A** and **B**) miR-195 inhibitor rescues proliferation (A), migration and invasion (B) reduced by CYTOR depletion in A549 cells. (**C** and **D**) miR-195 inhibitor rescues the IR-induced loss of cell viability (C) and the apoptosis (D) enhanced by CYTOR knockdown in A549 cells. (**E** and **F**) Transfection of miR-195 mimics suppresses proliferation (**E**), migration and invasion (**F**) increased by CYTOR overexpression in H1299 cells. (**G** and **H**) Transfection of miR-195 mimics suppresses the IR-induced loss of cell viability (C) and the apoptosis (D) decreased by CYTOR overexpression in H1299 cells. Significantly different from control (*P<0.05).

Based on the regulatory role of CYTOR on miR-195 expression, we further investigated the effect of CYTOR-miR-194 axis on proliferation, migration, invasion and radiosensitivity of NSCLC cells. A549 cells with CYTOR knockdown were transfected with miR-195 inhibitor. We found that the effects of CYTOR knockdown on proliferation, migration, invasion and radiosensitivity were reversed by miR-195 inhibitor in A549 cells (Figure 6A–D). Moreover, overexpression of wild-type CYTOR, but not CYTOR-mut, increased the proliferation, migration, invasion and radiotherapy resistance of H1299 cells, while miR-195 overexpression abolished these effects (Figure 6E–H).Taken together, the results revealed that the CYTOR regulates malignant phenotypes of NSCLC cells by sponging miR-195.

## Discussion

The present study highlights lncRNA CYTOR’s function and mechanisms in regulating NSCLC progression. CYTOR acts as a novel putative onco-lncRNA, which was overexpressed in many cancer types and promoted growth and metastasis. Nevertheless, the exact roles of CYTOR in NSCLC remain unknown. In the present study, we showed that CYTOR was significantly up-regulated in NSCLC tissues, and high-level CYTOR expression correlated with larger tumor size, advanced TNM stage, lymph node metastasis and poor prognosis, suggesting that CYTOR can be a strong predictor for NSCLC progression. Moreover, we demonstrated that CYTOR promoted NSCLC proliferation, migration and invasion, while suppressed radiosensitivity *in vitro*. Taken together, CYTOR can be a promising potential biomarker for prognosis and therapeutic target in NSCLC.

Endogenous transcripts containing microRNA response elements (MREs) can interact with or co-regulate each other by acting as endogenous microRNA sponges or ceRNAs, thereby forming regulatory network across the different transcriptome [25]. LncRNAs harbor potential MREs and function as ceRNA for microRNAs [26]. ceRNA regulatory networks are implicated in numerous biological processes in cancer, including tumorigenesis, epithelial–mesenchymal transition (EMT) and the invasion–metastasis cascade [17,27]. For instance, lncRNA ATB promotes the EMT process in liver cancer by regulating the miR-200s/ZEB1/2 pathway [17]. The novel lncRNA HCAL promoted hepatocellular carcinoma progression by sponging miR-15a/196a/196b and suppression of LAPTM4B expression [27]. CYTOR also plays a role in the regulation of many cancer types by acting as a ceRNA [28–30]. Based on the results of our bioinformatics analysis, it was hypothesized that miR-195 may be regulated by CYTOR. RIP, luciferase reporter and RNA pull-down assays were used to validate the interaction between CYTOR and miR-195. miR-195 targets were also increased by CYTOR. In addition, our findings revealed that the levels of CYTOR in NSCLC tissues were inversely correlated with that of miR-195. Finally, the effects of CYTOR on NSCLC cells depended on miR-195.

Most of NSCLC patients are diagnosed at an advanced stage, thus leading to a high mortality of NSCLC. High-energy radiotherapy and thoracic radiotherapy are currently the main nonsurgical approach for advanced NSCLC patients. However, the efficacy of radiotherapy is limited due to acquired radioresistance in the treatment of NSCLC. Therefore, identifying novel therapeutic targets to attenuate radioresistance is helpful for improving the outcome of NSCLC patients. Previous studies demonstrated that lncRNAs regulate the radiosensitivity of NSCLC. For example, PVT1 knockdown enhanced radiosensitivity of NSCLC cells by suppression of miR-195 [31]. Yang et al. [32] reported that lncRNA GACAT3 enhanced the sensitivity of NSCLC cells to radiotherapy. Our present study is the first to demonstrate the role of lncRNA CYTOR in radiosensitivity of NSCLC cells.

In conclusion, the present study demonstrated that CYTOR exerts as an oncogene in NSCLC progression. CYTOR enhances proliferation, migration and invasion, and alleviates the radiosensitivity in NSCLC cells *in vitro* by sponging miR-195. The present study revealed the underlying mechanism of CYTOR in NSCLC and may lead to novel therapeutic strategies for NSCLC.

## Abbreviations

CCK-8: Cell counting kit-8
CYTOR: cytoskeleton regulator RNA
EMT: epithelial–mesenchymal transition
LncRNA: long noncoding RNA
MRE: microRNA response element
NSCLC: nonsmall cell lung cancer
TNM: tumor, lymph node and metastases
